# State Medicine, Public Health, Hygiene and Bacteriology

**Published:** 1895-09

**Authors:** Ernest Wende

**Affiliations:** Health Commissioner of the City of Buffalo, N. Y.


					﻿STATE MEDICINE, PUBLIC HEALTH, HYGIENE AND
BACTERIOLOGY.
Conducted by ERNEST WENDE, M. D.,
Health Commissioner of the City of Buffalo, N. Y.
A Study in Vital Statistics.
By FRANKLIN C. GRAM, M. D., Registrar.
AN EXAMINATION of the statistics filed with the health
department of Buffalo is always of deep interest. I
have summarised a few of the principal ones for the first seven
months of the current year, and for the sake of study have com-
pared them with those of the same months for last year.
One of the first and most interesting features to note is the
fact that the death-rate is still diminishing, notwithstanding the
fact that this city is growing rapidly. In 1891, when the popula-
tion of Buffalo was 255,664, there were 6,001 deaths, making the
rate 23.48 per 1,000 ; in 1892 the rate was lowered to 19.98, in
1893 to 19.03, and in 1894 to 16.76. At the rate we are going
this will be considerably lowered in 1895, unless the unexpected
happens.
The following table gives some of the figures in which the
medical profession is mostly interested :
-	•	—• Fl Fl M £D '•'	~ ft! £»'	-	“1	3
£ £ ^ 5 S	H
x ce _	”*n‘ErCCjJ'J
-	-	»-^ »—« »—i ^^ ►—*	- - oj rj ~ “
^.m^XXOoOCOO^^	"i “<	^5	*5
o O	•*•	Ln 4m	<1	30 X
--....-	’	'	^ f x x £ £
...............................:••••■	g S
bO Cu
XOLnCnU.'	LmLm4*Cm-U	-U	4^	Cm CM	-U	Tntul	Hoathc
C ^3 C- "J Ln	O O U- Ln Cj	•-	-U	0	0^3	O	1 Oiai	^eainS.
-U oo 4^ Ln 4-	« X Q t-t -U	O	~	O	X h)	O
m m	)-(
O «-«CMLn4*^3L*J’^3Cn'*4LnLn4*i	C\	T,
•	•	Rate Der i.ooo.
0'04^00LnLuU-XO\LnO»-'CM	H ’
X	O	0	4^	~	LJ	^3	CX-P--U	XKJM	C^
Under five
C	IM	-•	Cm	Ln	4-	-4*	O	Lm	U Ln Ln	4. 4m 4m	lm	Voo^e
C>	—	N	—	w	O	00	00	cn	mi — Lm	O O lO	k>	lears.
Deaths from
m o	i!n >■ vo \q _ o o O-u O oo w o Communicable
□o-t-'Ouj im Ln o oo s> mj o ci o C\ o vo	Diseases.
_	—	_	Cholera infan-
O x o o n m	"	tniT1
LnOLMOMjM.«UK>—	M « LM im M	lU1IL
xocLn-u-ULcLM-t-LM-uLc-u-t*	u -U lm Consumption.
4-0	KI	Lm	4-	00	—	O	Ml	4-	Cl	L0	O	mJ	4-	vO
Ca> <->	Diphtheria
C. O	4-	—	-U	“	Lj	KJ	4*	M.	Ln	LJ	4-	lm	mJ	Ln	Renorted
c -	mj	00	00	00	O	-	Lm	—	X	mJ	X	Lm	MJ	o	neponeu.
J Diphtheria
O	Ci	mm	>-, mm	lj	mi	I	Deaths
Lm	\O	Lm	o	O Cl Cl	IM	O	O	MJ	OLniO	KJ	lm	|	mcius.
w -u	im Scarlet Fever
Ln	IM	IM	Ln	MC1IM	C\	K	Cl	Lm	MJ Ln oo	MI	o	Rpnortpd
O Im Cl — mJ X Ln m> X 4- mJ kJ X Ln X Ln	1	’
—	mj ' im im im	w w Scarlet Fever
Ln -u	o«“rMLMK>4MMjksooLMOK>C' Deaths.
oo	oi	-	« M »	x	Cl	g?	im „ kj - Typhoid Re-
KJ LMOCM IM Ln Ln LJ 1O 1O Cl O Ln w Ci KJ	ported.
10	O	-	M	M	4M	TyPr!?OidV,
m	n)	>o	Oh)	»-4*c^Ln,MOcncw	x	Ln	h)	Deaths.
«	cm ^3 o h)	Measles Re-
cm	*mJ	h)	« o	tj	»-<	^3	«	norted
\O	O	O	O —	~ O CC h) ~4-M O	L-	4-	-U	pvucu.
o	»-<	• cu	h) ’	«	Measles
C'Lncn»-<x>c~»-«Lw< o c\ m »-< w Deaths.
It will be seen that during the first seven months the death
record is 274 in favor of 1895, and that the number of deaths of
children, under five years, has also diminished. But the ratio of
infant mortality to that of adults is still too large, and this subject
alone is worthy of serious consideration. Likewise, there will be
noticed a decrease in the number of deaths from communicable
diseases, although there is some difference in cholera infantum.
The death-rate from consumption is remarkably close to that of
last year when numbers are considered. Although the percentage
in the total number of deaths is greater, yet the rate per 1,000 of
population is much smaller when we consider the city’s growth.
The medical profession is deeply interested in the next two
columns, showing the number of cases of diphtheria reported and
the proportion of deaths from the same. The assistance which
the department gives the profession by making microscopical
examinations in suspected cases, has had a tendency to make the
reports more accurate and also to indicate the true state of affairs
at an early stage of the illness. How much of the decrease in the
death-rate from this disease is due to the use of antitoxin cannot
be definitely stated, but from the reports received from practi-
tioners at various periods, there is no doubt but that this remedy
has had a great influence in the matter. The very large decrease
in the death-rate from scarlet fever would indicate that this disease
has not been so virulent as formerly. Everyone remembers the
epidemic of typhoid in the Spring of 1894, yet the percentage of
deaths to the number of cases was less than this year. It would
appear that there are few children left who have not had the
measles, judging from the number reported, and there ought to be
an immunity from this source for some time to come.
No comparative statement of existing cases of consumption
can be given, as it is but a short time since physicians have been
requested to report them. There were sixty cases reported during
the month of June and eighty-four in July.
Stop Milk-food.—In the summer diarrhea of infants and young chil-
dren, milk-food should be interdicted. Mere sterilisation of the milk
given is not sufficient. Fermentation may occur in a septic stomach or
intestine. The intestines should be cleansed, milk-food stopped and
antiseptic and sedative medication instituted. After a few days of this
treatment sterile milk may be given, as the alimentary canal is then in
a condition to bear it.—Polyclinic.
				

